# A Porcine-Based Task Trainer for the Instruction of Seidel’s Test and Ocular Foreign Body Removal

**DOI:** 10.7759/cureus.30190

**Published:** 2022-10-11

**Authors:** Nickolas E Srica, Ryan Walsh, Joseph Sikon, Lawrence Stack

**Affiliations:** 1 Department of Emergency Medicine, Vanderbilt University Medical Center, Nashville, USA

**Keywords:** emergency medicine training, emergency ophthalmology, ocular foreign body, simulation trainer, trainer, medical simulation, open globe, seidel’s test

## Abstract

Ophthalmologic emergencies account for tens of thousands of patients presenting to the emergency department (ED) each year. Studies continue to show that ophthalmology education in medical school is limited, and this leads to many resident physicians who lack confidence in their ophthalmological examination and procedural skills if opportunities to practice these skills are not deliberately sought out during their training. Medical simulation continues to be an excellent way for physicians to learn and master these anxiety-inducing procedures. We present a newly modified, innovative, and realistic porcine-based emergency ophthalmologic procedural task trainer with live television screen projection to further improve Emergency Medicine (EM) resident education on the performance of Seidel’s test and ocular foreign body removal.

## Introduction

Ophthalmological complaints make up a significant number of Emergency Department (ED) visits each year in the United States (US). One recent study looking at data from the US Nationwide Emergency Department Sample showed that from 2006 to 2011, there were nearly 12 million ED visits for ocular problems, of which 36.3% were due to eye injuries, and another 7.5% were due to ocular foreign bodies [1]. Studies and surveys also continue to show that formal ophthalmological education and procedural training are lacking in medical education [2,3]. Gelston and Patnaik showed in their study that a large proportion of Emergency Medicine (EM) and other resident physicians lack confidence in their ophthalmological examination skills. However, they also found that increasing the time spent practicing these competencies appeared to directly lead to increased confidence in caring for patients with ocular conditions [4]. To be prepared for the majority of ophthalmologic emergencies presenting to the ED, EM residents should master the performance of a proper slit lamp exam, Seidel’s test, tonometry, and ocular foreign body removal.

## Technical report

We sought to modify and innovate on our previously described high fidelity, porcine-based emergency ophthalmological procedural task trainers to further improve EM resident education on the performance of Seidel’s test and ocular foreign body removal [5]. We have modified and improved the trainer to now include connecting the slit lamp to a large television screen in order to provide real-time feedback to the learner as well as keep all other learners engaged.

Briefly, to create the task trainer, swine eyes were obtained from a local meat processing business. Ocular muscles, lids, and orbital fat were trimmed from the globe. “Orbits” were created in styrofoam heads by simply removing excess styrofoam. Swine eyes were fitted into the “orbits” using oval-shaped gauze pads creating a pressure-friction dynamic that kept the eyes in place. The styrofoam head was then secured in the slit lamp with a Velcro strap.

For Seidel’s test, a 1.5-inch 22-gauge needle was placed through the lateral aspect of the styrofoam orbit and into the globe. This was secured to the head with tape. IV start kit tubing was placed onto the needle and attached to a 5mL syringe filled with saline. A 25-gauge needle was used to puncture the cornea at a perpendicular angle near the visual axis. Once removed, this puncture into the anterior chamber results in an open globe injury. The amount of leakage through this puncture site can be controlled by depressing the plunger on the 5ml syringe. Residents are then taught to use a moistened fluorescein strip under cobalt blue light to assess for an ocular leak of fluid (Seidel’s test) typically seen in an open globe injury.

For ocular foreign body and rust ring removal, a nail was placed against a grinder in close proximity to the swine eye. This resulted in numerous metallic foreign bodies. Rust rings were simulated by placing pinpoint markings with a bronze-colored permanent marker on the cornea. Learners were then taught how to properly use a small gauge needle to remove foreign bodies from the surface of the eye, as well as how to use the Algerbrush for rust ring removal.

**Figure 1:**
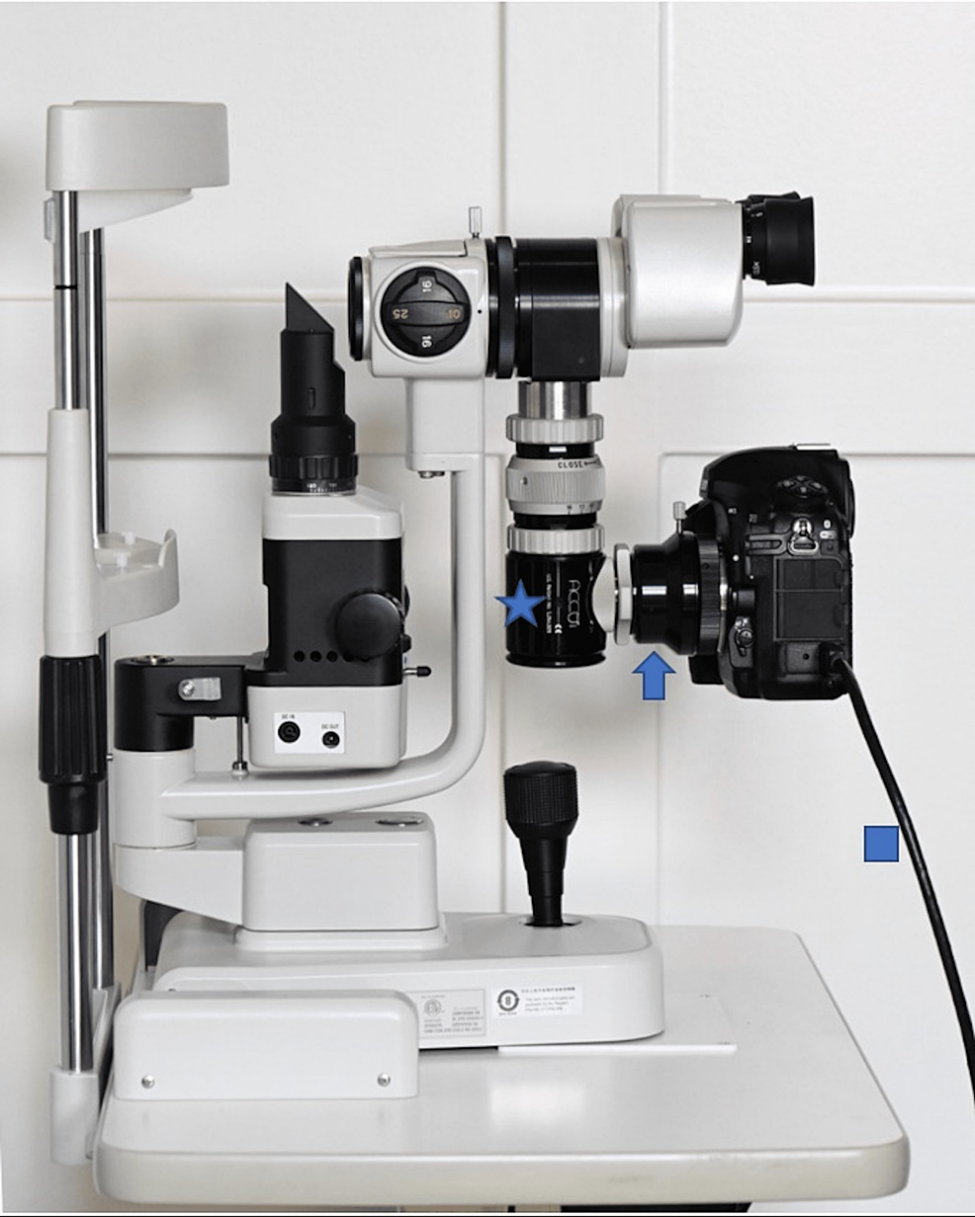
Enlarged image showing the full slit lamp setup, including digital single-lens reflex camera (far right), TTI Medical Accu-Beam® Digital SLR Adapter (arrow) and Accu-Beam® Universal C-mount Video Adapter (star), and HDMI cable connection from camera to television for the projection (square).

**Figure 2:**
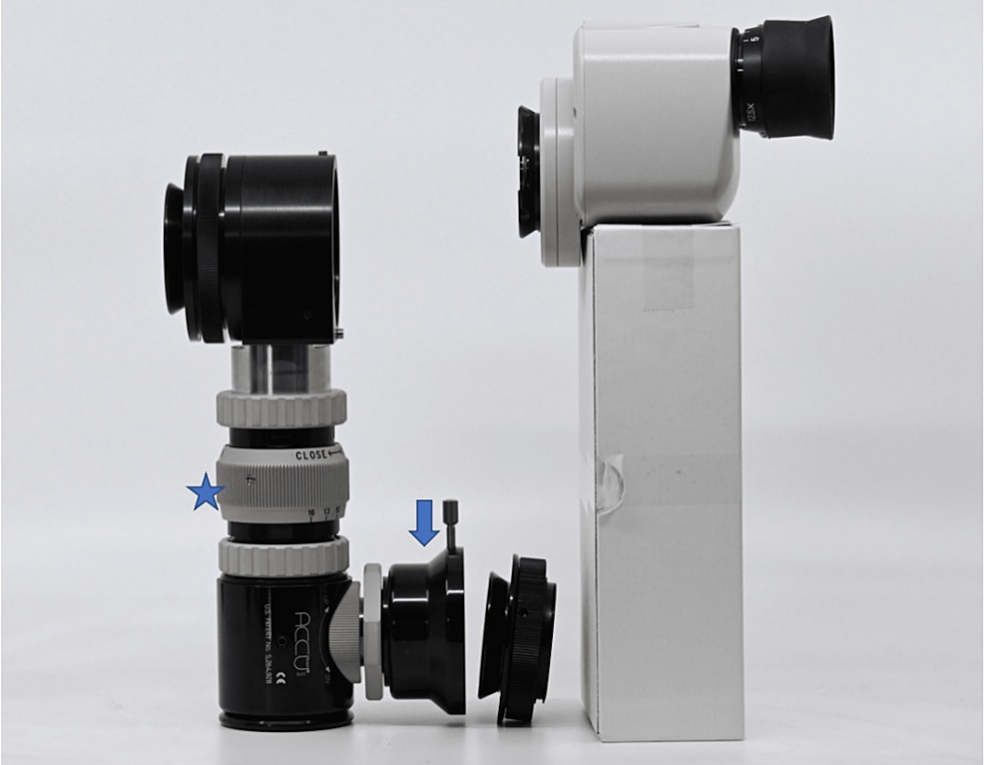
TTI Medical Accu-Beam® Digital SLR Adapter (arrow) and Accu-Beam® Universal C-mount Video Adapter (star).

**Figure 3:**
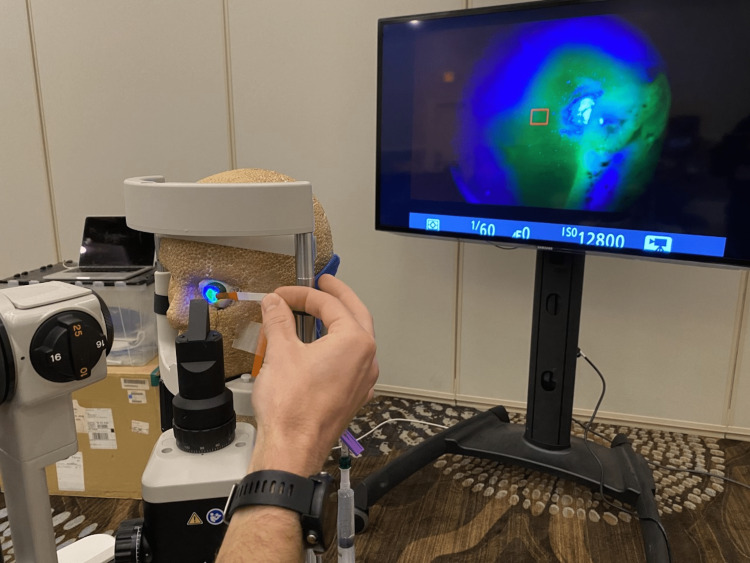
Seidel’s test being performed on our task trainer with television screen projection of slit lamp.

An essential component to properly performing both of the above ophthalmological skills is first gaining confidence with the slit lamp exam. Without previously being able to see exactly what the learner was seeing, we as educators were unable to feel completely confident in the skill level of the learner. We also found it difficult to provide constructive real-time feedback for improvement. To address this limitation, we found that connecting a digital single-lens reflex camera in “live view” video mode to the slit lamp itself remedied this situation. We did this by connecting the camera to the slit lamp itself via a TTI Medical Accu-Beam® Digital SLR Adapter (TTI Medical, San Ramon, CA) and a TTI Medical Accu-Beam® beamsplitter (TTI Medical). The camera was then connected directly to a large television screen using an HDMI cable (Figures 1, 2) allowing all present learners to visualize the slit lamp view. This enabled far more individualized and useful real-time guidance (Figure 3). An additional benefit of this setup was that the learner performing the procedure was still able to utilize the slit lamp as they normally would without any interference from the camera adapter.

## Discussion

A total of 22 PGY 1-3 EM residents completed the training session, and all present learners completed an anonymous post-course survey in which they rated aspects of the session on a 5-point Likert scale. Descriptive statistics of this data were then performed.

The use of this task trainer during our annual emergency ophthalmology education day and during one of our monthly simulation sessions has been extremely well received by all participating residents. Unanimously (22/22), the participating residents stated that they “strongly agreed” (rated 5 on a 5-point scale) that this was an overall valuable educational experience and that the task trainer model utilized was adequate to learn the necessary procedural skills. Residents also rated their overall comfort in performing these emergency ophthalmologic procedures after this training very highly (mean 4.32, SD 0.65).

**Figure 4:**
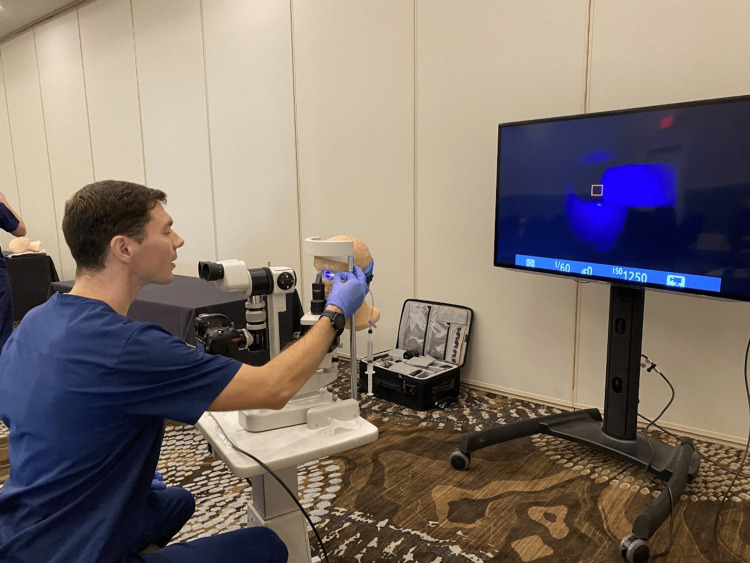
Seidel’s test being performed on our task trainer with a television screen projection of slit lamp.

Ocular complaints continue to make up a significant portion of ED visits, and it is essential that EM physicians are confident in their examination and procedural skills in these often urgent or emergent situations [6]. This additional innovation to our prior ocular educational sessions received very favorable feedback, and we found that it assisted us as educators as we could see in real-time what the learner saw through the slit lamp biomicroscope enabling us to provide real-time guidance to the learner. Additionally, it kept the other learners engaged while they waited for their turn for hands-on experience, while also allowing them to visualize the techniques of others to further hone their own skills (Figure 4).

## Conclusions

Given the overall lack of confidence most EM resident physicians describe in their ability to perform many emergency ophthalmological procedures, it is essential that educators continue to develop safe, practical, and fun ways for resident physicians to develop confidence and competence with these procedures. This task trainer in conjunction with the television screen projection innovation is one that could easily be adopted by other EM programs to further improve resident education on core ophthalmological procedural competencies.
